# Healthy Vision Month — May 2015

**Published:** 2015-05-22

May is Healthy Vision Month, a national observance devoted to encouraging persons to make vision and eye health a priority. CDC’s Vision Health Initiative partners with the National Eye Institute’s National Eye Health Education Program to educate the public about vision loss prevention and eye health promotion.

Early detection, timely treatment, and use of protective eyewear are the best ways to maintain eye health and prevent or delay vision impairment, defined as the best-corrected visual acuity <20/40 in the better-seeing eye (1). Nearly 38 million persons in the United States have common eye diseases such as glaucoma, diabetic retinopathy, age-related macular degeneration, and cataracts (2).

Regular, comprehensive, dilated eye examination is the only way to detect vision problems and eye diseases in their early stages. The American Academy of Ophthalmology and American Optometric Association recommend regular, comprehensive, dilated eye examination to detect vision problems and eye diseases in their early states for all persons aged ≥65 years and younger persons with diabetes or risk factors for glaucoma (3,4). Additional information about vision and eye health is available at http://www.cdc.gov/visionhealth and http://www.nei.nih.gov/healthyeyes.
